# Recent insights from in vitro single-molecule studies into nucleosome structure and dynamics

**DOI:** 10.1007/s12551-016-0212-z

**Published:** 2016-10-18

**Authors:** Orkide Ordu, Alexandra Lusser, Nynke H. Dekker

**Affiliations:** 1 Bionanoscience Department, Kavli Institute of Nanoscience,, Delft University of Technology, Van der Maasweg 9,, 2629 HZ Delft, The Netherlands; 2 Division of Molecular Biology, Biocenter, Medical University of Innsbruck, Innrain 80-82, 6020 Innsbruck, Austria

**Keywords:** Single-molecule techniques, (Sub)nucleosome structure and dynamics, Histones, Nucleosome remodeling, Post-translational modifications, Transcriptional barrier

## Abstract

Eukaryotic DNA is tightly packed into a hierarchically ordered structure called chromatin in order to fit into the micron-scaled nucleus. The basic unit of chromatin is the nucleosome, which consists of a short piece of DNA wrapped around a core of eight histone proteins. In addition to their role in packaging DNA, nucleosomes impact the regulation of essential nuclear processes such as replication, transcription, and repair by controlling the accessibility of DNA. Thus, knowledge of this fundamental DNA–protein complex is crucial for understanding the mechanisms of gene control. While structural and biochemical studies over the past few decades have provided key insights into both the molecular composition and functional aspects of nucleosomes, these approaches necessarily average over large populations and times. In contrast, single-molecule methods are capable of revealing features of subpopulations and dynamic changes in the structure or function of biomolecules, rendering them a powerful complementary tool for probing mechanistic aspects of DNA–protein interactions. In this review, we highlight how these single-molecule approaches have recently yielded new insights into nucleosomal and subnucleosomal structures and dynamics.

## Introduction

The genome of eukaryotic organisms ranges from millions to hundreds of billions of base pairs for different species and can be stretched accordingly to millimeters or meters (Kidwell 2002; Merhej and Raoult 2012; Zimmer 2007). These lengths are several orders of magnitude larger than the cell nucleus, with an average diameter of roughly 5 μm, and therefore the genome must be tightly packed in order to fit into the nucleus. To achieve an appropriate compaction level, eukaryotic organisms organize their genome in hierarchical protein–DNA assemblies termed chromatin that inevitably influence DNA accessibility during key cellular processes. Hence, revealing the details of chromatin structure is essential for understanding the regulation of the genome. A major milestone was set about forty years ago, when chromatin was first reported to comprise a repeating unit of ∼200 bp of DNA wrapped around a core of histone proteins (Kornberg 1974; Olins and Olins 1974; Oudet et al. 1975). This basic component of chromatin, termed the nucleosome, has been a central subject of research ever since.

Bulk studies based on traditional biochemical approaches from molecular and structural biology have provided invaluable insight into nucleosome structure and function (Kornberg 1977; Kornberg and Lorch 1999; Li et al. 2007; McGhee and Felsenfeld 1980; Workman and Kingston 1998; Zentner and Henikoff 2013). However, these methods only reflect collective properties of samples due to ensemble and time-averaging. Partial features, such as rare or transient events, and especially the intrinsic dynamics of usually heterogeneous biological samples cannot be resolved.

When many identical biomolecules are present in a solution, each one can occupy any one of a number of different conformational states with similar energies. Assuming for simplicity that each state corresponds to a distinct, visible color, the whole solution will at any given time display a color that reflects the average of all the individual molecules, which will each occupy different states with their corresponding distinct colors. Likewise, the observation of a single molecule on a long timescale, during which the molecule can convert between all states with the corresponding colors, will also yield an average color. The heterogeneity arising from both static differences between single biomolecules and individual dynamic changes in, for example, their structure, function or chemical modification remains therefore hidden.

Details of molecular properties can be revealed by directly studying single molecules over time. However, the insights that can be obtained here strongly depend on the characteristics of the experimental system in use and the spatial and temporal resolution of the applied technique. During the observation within a certain time frame, the molecule might convert between barely different states at such high speed that intermediates would be averaged out and obscured. Hence, since the 1990s the development of single-molecule studies has not only included the interrogation of different biological systems but also the continued improvement of their accuracy and sensitivity (Dulin et al. 2013; Eigen and Rigler 1994; Hell 2009; Huang et al. 2009; Jares-Erijman and Jovin 2003; Joo et al. 2008; Moerner 2007; Neuman and Nagy 2008; Tinoco and Gonzalez 2011; Weiss 1999). Single-molecule techniques have become a highly suitable tool for investigating DNA–protein interactions at the molecular level (Duzdevich et al. 2014; Heller et al. 2014; Monico et al. 2013). In the context of DNA compaction into chromatin, they have also yielded new, complementary findings on the structure, function and especially dynamics of chromatin and nucleosomes (Buning and van Noort 2010; Choy and Lee 2012; Killian et al. 2012; Lavelle et al. 2011; Petesch and Lis 2012; Zlatanova and Leuba 2003).

In this review, we will introduce the most commonly used single-molecule techniques in nucleosome research and present the recent insights they have provided into nucleosomal and subnucleosomal structure, function and dynamics within the last 3 years.

## Single-molecule techniques in nucleosome research

Over the past two decades, time-resolved observation and manipulation of single molecules have become very powerful means to investigate biological systems. The major single-molecule techniques enable the researchers to either directly visualize or influence individual molecules to reveal molecular details of their structure, function and dynamics on the nanometer scale. Substantial technical advances in optical microscopy and fluorescent probes have made fluorescence microscopy and fluorescence spectroscopy routine methods for directly visualizing and observing single molecules over time (Giepmans et al. 2006; Moerner and Fromm 2003). The manipulation of individual molecules using force spectroscopy has become possible by the development of trapping methods with different types of force transducers (Neuman et al. 2007). Among these techniques, atomic force microscopy (AFM) is a unique method that enables either the direct observation or the manipulation of single molecules by imaging or trapping, respectively (Butt et al. 2005; Hansma and Hoh 1994). The most commonly employed single-molecule techniques and their associated specific experimental approaches are described in the following subsections.

### Fluorescence microscopy and fluorescence spectroscopy

Fluorescence microscopy essentially relies on the detection of light emitted at a specific wavelength by specific molecules (fluorophores) that are fused to the biomolecule of interest following their excitation at an initial, typically shorter, wavelength. The type and precise characteristics of these fluorophores will ultimately determine the efficiency and applicability of this technique to a specific study (Berlier et al. 2003; Shaner et al. 2005). Fluorescent samples can be excited either in a large or a small area depending on the design of the microscope. In wide-field microscopy, the sample is illuminated by a nearly collimated light beam, resulting in the simultaneous excitation of numerous fluorophores at different depths of focus and therefore in considerable background noise (Funatsu et al. 1995; Schmidt et al. 1996). This out-of-focus fluorescence is substantially decreased in total internal reflection fluorescence microscopy (TIRFM), which restricts the illumination depth to about 100 nm via a highly localized, quickly decaying electromagnetic field (evanescent field) that is generated at the sample surface (Axelrod 1981, 2001). Confocal microscopy reduces the excitation volume and almost entirely eliminates out-of-focus light by using a focused laser beam and a spatial pinhole positioned just before the detector (Minsky 1988; Nie et al. 1994). These excitation methods are used in combination with different fluorescence spectroscopy techniques depending on the research question.

**Fig. 1:**
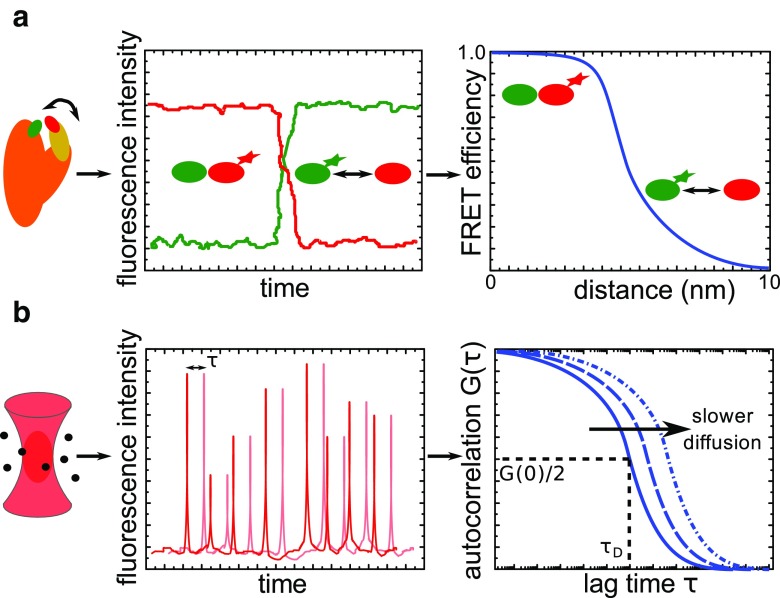
Sketched examples of fluorescence spectroscopy techniques. **a** Single-molecule fluorescence resonance energy transfer (*smFRET*). A dynamic molecule (*orange*/*yellow*) is labelled with a donor (*green*) and an acceptor (*red*) fluorophore. When the fluorophores are close together within 10 nm, the excited donor will transfer its energy to the acceptor for fluorescence emission. For distances of >10 nm, only the donor will fluoresce. The recorded time-resolved fluorescence signals of the donor (*green*) and acceptor (*red*) are used to calculate the efficiency of FRET. **b** Fluorescence correlation spectroscopy. Very few molecules (*black*) diffusing through the tiny excitation volume generated in a confocal microscope are excited for fluorescence emission. The time-resolved fluorescence signal is recorded and analyzed by autocorrelation, i.e. checking its similarity to its replicas shifted by different lag times (*τ*). The resulting autocorrelation function [*G(τ)*] yields the characteristic parameters of the underlying process, such as the diffusion time (*τ*_*D*_) at about half of the amplitude [*G(0)/2*]

Förster or fluorescence resonance energy transfer (FRET) is a well-established method to study the structural dynamics of single molecules. It is based on the non-radiative energy transfer between two fluorescent probes in very close proximity (Förster 1948; Ha et al. 1996). An excited fluorophore (donor) can non-radiatively transfer its energy to another, sufficiently close fluorophore (acceptor) which then emits fluorescent light (Fig. 1a). By monitoring this energy transfer and its efficiency in real-time, the dynamics and distances of inter- and intramolecular interactions involved in conformational changes can be revealed on the scale of 1–10 nm. This method was first applied to nucleosomes for studying their structural dynamics (Tomschik et al. 2005). The efficiency of FRET between a donor and an acceptor fluorophore each located on the nucleosomal DNA at ∼45 bp from its entry and exit sites showed fast dynamic changes in nucleosome structure between a long-lived, fully wrapped state (2–5 s) and a transient, substantially unwrapped conformation (100–200 ms). This work represents the first attempt to directly investigate the dynamic nature of nucleosomes, and the results suggest a potential mechanism by which DNA accessibility for DNA-binding proteins can be regulated. However, the observed dynamics was highly affected by fluorophore blinking, which influenced the results and was corrected for in a later publication by the authors (Tomschik et al. 2009). Different FRET-based assays have subsequently been developed and used, enabling more accurate, robust and reliable insights into nucleosome structure and dynamics (Blosser et al. 2009; Koopmans et al. 2007; North et al. 2012; Simon et al. 2011).

Another frequently used single-molecule fluorescence technique is fluorescence correlation spectroscopy (FCS), which allows the study of the dynamics of individual molecules as they freely diffuse in solution (Elson and Magde 1974; Magde et al. 1972, 1974; Rigler et al. 1993). FCS is based on the correlation analysis of fluctuations in the time-resolved fluorescence signal that arises from very few molecules diffusing through the tiny excitation volume (∼10^−3^ pl) generated in a confocal microscope (Fig. 1b). Essentially, the fluorescence signal is compared to its replicas calculated at different lag times to check their similarity and reveal repetitive patterns due to the underlying physical processes, such as free diffusion, chemical reaction or conformational changes. The resulting mathematical expression (the autocorrelation function) yields the characteristic parameters of these processes, such as diffusion constants, concentrations, hydrodynamic radii or reaction rates. FCS was first used to investigate the structural dynamics of nucleosomes in combination with FRET (Li et al. 2005). The un- and rewrapping rates of the nucleosome were initially determined indirectly via FRET by trapping the open conformation using a site-specific DNA-binding protein. FCS measurements were then performed on nucleosomes labeled with either donor only or a donor–acceptor pair to directly observe conformational changes for a more reliable interpretation of the kinetics. The results obtained from both approaches led to the conclusion that nucleosomal DNA unwraps on a timescale of ∼250 ms and rewraps more rapidly within 10–50 ms. Subsequent efforts using this technique have provided additional insights into nucleosome structure and dynamics (Bohm et al. 2011; Gansen et al. 2009; Koopmans et al. 2009; Poirier et al. 2009; Tims et al. 2011).

These studies convincingly demonstrate the great power of the most commonly used single-molecule fluorescence techniques to visualize DNA–protein interactions. When used in combination, they can yield complementary insights that allow the researchers to draw more reliable conclusions. However, the data acquisition, analysis and interpretation in fluorescence microscopy studies must always take into account the many factors related to the photophysics of the fluorophores (Ha and Tinnefeld 2012). This issue is entirely eliminated in force spectroscopy techniques which rely on the detection of light scattered by micron-sized massive particles. The most common approaches of these manipulation methods are presented below.

### Force spectroscopy

The basis of force spectroscopy techniques is the specific attachment of single molecules between a substrate and a force transducer by interacting proteins or organic or engineered compounds. This tethering enables manipulation of the molecules by the application of forces and, in some cases, torques. The readouts provided by the force transducers are tracked in real-time, allowing for the investigation of primarily mechanical properties of the sample. Depending on the biological question of interest, different force transducers are used together with distinct methods for trapping and monitoring them.

**Fig. 2:**
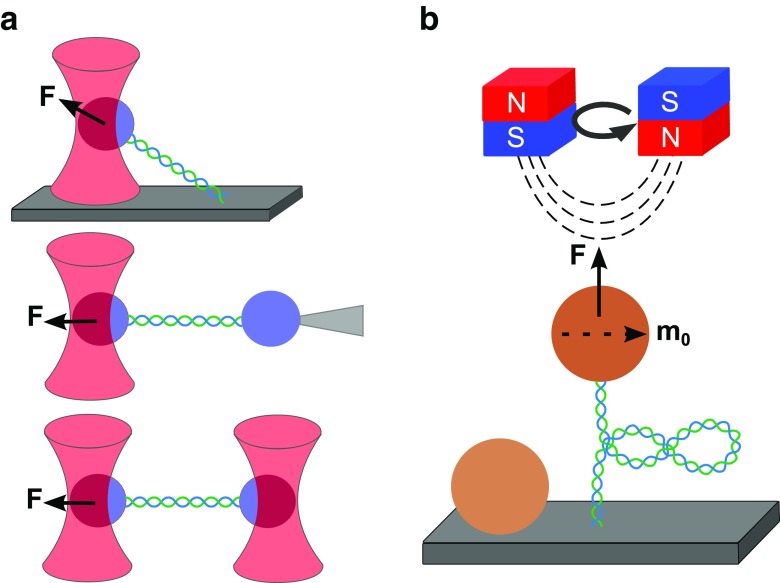
Overview of force spectroscopy techniques. **a** Optical tweezers. A DNA molecule (*blue/green*) is tethered between an optically trapped dielectric microsphere (*violet*) and either the glass coverslip (*top*) or another bead fixed using a micropipette (*middle*) or a second optical trap (*bottom*). Moving the optical trap will change the tether’s extension and tension related to the applied forces (*F*), ranging between 0.1 and 100 pN (extension clamp). **b** Magnetic tweezers. A DNA molecule is tethered between the glass coverslip and a magnetic bead that is trapped using a pair of cubic permanent magnets (*red/blue*) which accurately exert forces ranging between 10^-3^ and 100 pN due to the magnetic field gradient (force clamp). Due to an induced horizontal magnetic moment (*m*_*0*_), the bead is also torsionally trapped, which allows the application of torques by rotating the permanent magnets. Torque application leads to supercoiling of the DNA molecule and the formation of plectonemes (*circles of DNA*). Non-magnetic reference beads adhered to the surface are used to correct for drift

In optical tweezers (OT) dielectric micron-sized particles are captured in the focus of an intense laser beam exerting a force due to the light gradient (Ashkin 1992; Ashkin et al. 1986; Smith et al. 1996). In biological applications, OT typically involve a nucleic acid molecule tethered between an optically trapped bead and a substrate, which can either be the surface of the sample holder or another, fixed bead held by a micropipette or even a second optical trap (Fig. 2a). The underlying mechanism is based on controlling the position of the trapped bead and, thereby, the molecule’s extension. Therefore, this type of trapping is referred to as an extension clamp. Moving the focused laser beam with the trapped bead allows the manipulation of the molecule by inducing a concomitant change in its extension, which concurrently affects the molecule’s tension that is related to the applied forces ranging between 0.1 and 100 pN. The bead’s position is recorded indirectly by detecting the laser signal on a position-sensitive device, which enables the simultaneous measurement of force and extension, the two key quantities of force spectroscopy. This method was first used in chromatin research to study the structure of native chromatin fibers extracted from chicken erythrocytes (Cui and Bustamante 2000). The mechanical stretch–release manipulation revealed a reversible decondensation of the fibers at low forces (<6 pN), which was attributed to internucleosomal interactions. Specifically, the fibers showed a pronounced transition between condensation and decondensation at 5–6 pN under physiological salt concentrations, indicating strong internucleosomal interactions with energies comparable to the thermal energy. Upon pulling at high forces (>20 pN), the fibers were observed to undergo irreversible changes in their extension, which was explained by the possible eviction of the histone proteins during this mechanical unfolding. The results of this study led to the first insights into the energy landscape of chromatin structure and also suggested a considerable dynamic nature due to thermal fluctuations. It was followed by many other OT-based assays that shed more light on the structure and dynamics of nucleosomes (Bennink et al. 2001; Bintu et al. 2012; Brower-Toland et al. 2005; Gemmen et al. 2005; Hall et al. 2009; Hodges et al. 2009; Jin et al. 2010; Mack et al. 2012; Pope et al. 2005; Shundrovsky et al. 2006; Sirinakis et al. 2011; Zhang et al. 2006).

Another very common technique used for mechanical manipulation of single molecules is magnetic tweezers (MT). In this method, magnetic beads are trapped by permanent or electrical magnets that exert a force as a result of a magnetic field gradient (Amblard et al. 1996; Crick and Hughes 1950; Smith et al. 1992; Strick et al. 1996, 1998; Ziemann et al. 1994). In the most common designs a nucleic acid molecule is tethered between a magnetic bead and the surface of the sample holder at its two extremities and manipulated using permanent magnets (Fig. 2b). Vertical movement of the magnets results in a corresponding change of the applied force, ranging from 10^−3^ to 100 pN, and a concomitant change of the bead’s position, which is directly recorded by video microscopy with a charge-coupled-device (CCD) camera. As the applied force is the parameter that is precisely controlled in this technique, the underlying mechanism is also referred to as force clamp. However, besides forces, MT can also apply torques by rotating the magnets. MT were first used in chromatin research to study the time- and force-dependence of the assembly and disassembly of chromatin fibers (Leuba et al. 2003). Fibers were found to assemble only at forces up to 10 pN, while assemblies at the higher forces within this range were observed to be reversible. These results revealed a strong dependency of chromatin assembly on the force applied to the DNA and illustrated the dynamic equilibrium of this process. Translated to a possible scenario in the cell, these experiments allow conclusions to be drawn on the potential fate of chromatin/nucleosomes under forces exerted by enzymes during DNA-templated processes. For example, the forces generated by the *E. coli* RNA polymerase (RNAP) were shown to be in the picoNewton range (Wang et al. 1998). Subsequent MT studies confirmed and further refined these results to a more detailed picture of the structure and dynamics of chromatin and nucleosomes (Kruithof et al. 2009; Lia et al. 2006; Simon et al. 2011; Vlijm et al. 2012; Yan et al. 2007). The specific function of the nucleosome in higher-order folding of chromatin based on inter-nucleosomal interactions has also been assessed using single-molecule force and torque spectroscopy, but it still remains elusive due to additional restrictions on the electrostatics, topology and elasticity of the complex (Chien and van Noort 2009; Lavelle et al. 2010). While MT and OT have become the routine approaches for force spectroscopy due to their simple yet robust principles, they are, however, limited to mechanical manipulation of samples and do not allow direct observation.

### Atomic force microscopy

**Fig. 3:**
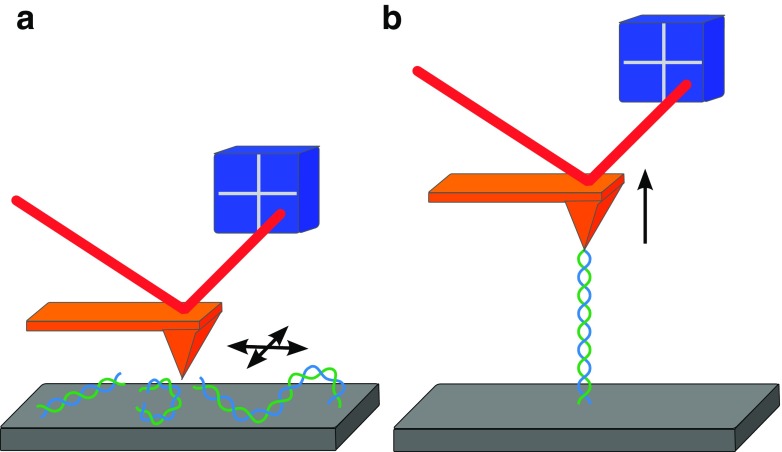
Principles of atomic force microscopy. A cantilever (*orange*) is used to exert atomic forces on the sample. Their interaction leads to distortions of the cantilever which is recorded using a laser beam (*red*) that is reflected onto a position-sensitive device such as a quadrant photodiode (*blue*). **a** The cantilever can scan the sample to obtain a topographical image. **b** A DNA molecule is tethered between the glass coverslip and the cantilever to exert forces between 10 and 10^4^ pN for force spectroscopy

Atomic force microscropy (AFM), also called scanning force microscopy (SFM), is a technique that is capable of either observing or manipulating single molecules on the same instrument by imaging or force spectroscopy, respectively (Binnig et al. 1986; Florin et al. 1994). Both principles are based on the use of a cantilever as the force transducer. This cantilever is either scanned over a sample to obtain a topographical image by means of atomic interactions or tethered to one extremity of an individual molecule for its mechanical manipulation. Its interaction with the sample involving forces ranging between 10 and 10^4^ pN leads to the bending of the cantilever, which is tracked by the use of a laser beam directed on the cantilever and reflected onto a position-sensitive device (Fig. 3). In this way, either the topology of a sample or the extension of a molecule can be indirectly read out with near atomic resolution (≤1 nm) by controlling the position of the cantilever. Hence, like OT, AFM also operates primarily as an extension clamp in force spectroscopy. In nucleosome research, this technique is mainly used for imaging, as the applied forces are in the higher range of the molecular scale and the distinct structures of chromatin, such as mono- and polynucleosomes, or higher-order foldings into fibers are very suitable to study using this specific approach. The first AFM study was performed on nucleosome arrays in order to directly observe and characterize their structural details (Allen et al. 1993). This work convincingly illustrated the applicability of AFM imaging for high-resolution studies on nucleosome structure and was followed up by many researchers investigating the dynamics, as well as the role of nucleosomes in DNA accessibility (Bintu et al. 2011; Dalal et al. 2007; Dimitriadis et al. 2010; Miyagi et al. 2011; Shlyakhtenko et al. 2009; Yoda et al. 2000). Therefore, AFM represents another widely used technique in nucleosome research in addition to fluorescence and force spectroscopy. In the following sections we highlight recent insights into (sub)nucleosomal structure and dynamics from studies using most of the specific single-molecule approaches presented here.

## Nucleosome structure and dynamics

**Fig. 4:**
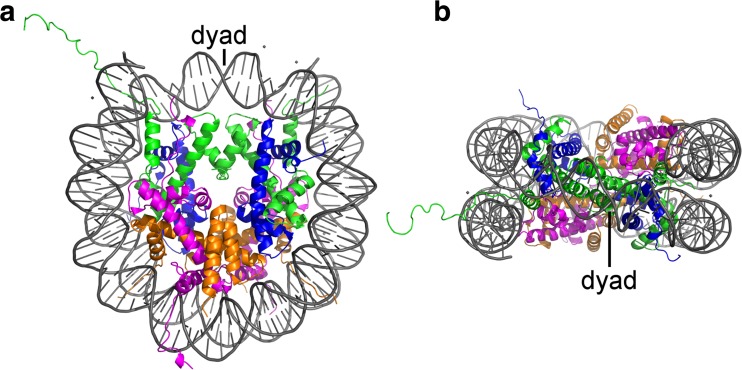
Structure of the nucleosome. A total of 147 bp of DNA (*grey*) are wrapped around a discoidal protein structure containing two copies of the eight core histones H2A (*magenta*), H2B (*orange*), H3 (*green*) and H4 (*blue*) in a left-handed superhelix. **a** Top view. **b** Side view along the pseudo-twofold symmetry (dyad) axis. The images were created from the structural data in the RCSB Protein Data Bank (PDB) with the identification code 1AOI (Luger et al. 1997) using the PyMOL Molecular Graphics System, Version 1.7.2.1 Schrödinger, LLC

The nucleosome consists of 147 bp of DNA wrapped ∼1.7 times in a left-handed superhelix around a discoidal protein structure of ∼5 nm in height and ∼7 nm in diameter formed by eight histones (Davey et al. 2002; Luger et al. 1997; Richmond et al. 1984) (Fig. 4). This histone octamer contains two copies of each of the so-called core histones H2A, H2B, H3 and H4 that are assembled into four heterodimers, i.e. two H2A/H2B and two H3–H4 dimers, by short-range interactions between the central α-helical histone-fold domains in a ‘handshake’ manner (Arents et al. 1991; Klug et al. 1980). The two H3–H4 dimers join to form a tetramer through the four-helix bundles of the H3 histones centered on the pseudo-twofold symmetry (dyad) axis, while the two H2A/H2B dimers attach to the tetramer via similar four-helix bundle interactions between the H2B and H4 histones. Each core histone further features a flexible N-terminal tail, while the histone H2A additionally exhibits a C-terminal tail. All of the histones are highly positively charged, and as such they balance the negative charge of the DNA. Hence, the histone octamer is only found to be stable in the presence of DNA or at high salt concentrations (∼2 M), and it dissociates into the (H3–H4)_2_ tetramer and the two H2A/H2B dimers at physiological conditions (Eickbush and Moudrianakis 1978). Likewise, the nucleosome is assembled in a stepwise manner by the initial binding of the (H3–H4)_2_ tetramer to the DNA and the subsequent incorporation of the H2A/H2B dimers (Polo and Almouzni 2006). However, the nucleosome complex resulting from this well-defined assembly pathway is not static, but a highly dynamic entity. Its inter-dependent structural, mechanical, chemical and functional properties are continuously altered by different mechanisms, such as intrinsic dynamics, chemical modifications of the DNA and histones, ATP-dependent remodeling, as well as by forces and torques exerted by genome processing enzymes. The concerted action of all of these mechanisms makes it very difficult to study this complex system as a whole using single-molecule techniques. Such methods can, however, provide invaluable insights into the different individual mechanisms and their impact on nucleosome structure, dynamics and function (Choy and Lee 2012; Killian et al. 2012).

### Intrinsic nucleosome dynamics

**Fig. 5:**
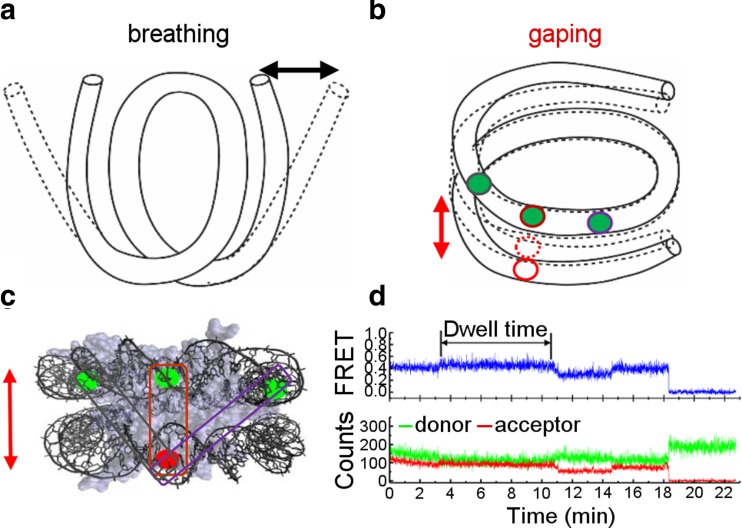
Intrinsic nucleosome dynamics. **a** The nucleosomal DNA ends transiently wrap and unwrap from the histone octamer (breathing), indicated by the *black arrow*. **b** The two turns of nucleosomal DNA transiently open with respect to each other along the superhelical axis (gaping), depicted by the *red arrow*. **c** Different labelling schemes to identify the gaping transition which is best characterized by the FRET pair encircled in red. **d** Time-resolved fluorescence signals of the donor (*green*) and acceptor (*red*) fluorophore from the best characterized FRET pair are recorded and yield the FRET efficiency, showing a slight increase over several minutes due to gaping. All panels (**a–d**) are figures reprinted with minor changes from Ngo and Ha (2015), Copyright (2015), used under a CC BY 4.0 license (http://creativecommons.org/licenses/by/4.0/). This figure is not included in the present article's Creative Commons license

The observation of nucleosomes at the single-molecule level have revealed that their structure is intrinsically dynamic. As mentioned above, about 30 bp at the entry and exit sites of the nucleosomal DNA were reported to spontaneously unwrap and rewrap on a timescale between 10–300 ms (Koopmans et al. 2007; Li et al. 2005; Miyagi et al. 2011) (Fig. 5a). However, until recently this phenomenon of DNA breathing was studied either indirectly with assays using DNA-binding proteins to trap the open nucleosome conformation or directly using methods limited in their specific time resolution for technical reasons. Very recently, the transient unwrapping and rewrapping of nucleosomal DNA ends has been identified on a timescale of 1–10 ms using a novel single-molecule technique combining single-molecule FRET (smFRET) and FCS with stochastic data analysis based on maximum likelihood estimation (MLE) (Wei et al. 2015). This approach enables the study of the structural dynamics of biomolecules on the sub-microsecond timescale considering the photophysical properties of the fluorophores. By this means, this study provided the first direct evidence that DNA breathing is a very fast process. In another recent work, the first experimental evidence for a novel spontaneous transition of nucleosome structure, called gaping, was reported using smFRET (Ngo and Ha 2015). This phenomenon refers to the transient opening of the two turns of nucleosomal DNA with respect to each other along the superhelical axis (Fig. 5b). Different labeling schemes were used to study this conformational change associated with an estimated distance change of 0.5–1 nm and a timescale of 1–10 min (Fig. 5c, d). However, due to technical limitations in terms of resolution owing to the use of FRET and the labeling strategy based on the use of a linker, further high-resolution studies are needed to reveal the details of this phenomenon, including potential structural changes in the histone octamer. Further evidence supporting these recent findings will certainly have strong implications for the role of the intrinsic structural dynamics of nucleosomes as a major mechanism for regulating DNA accessibility in the context of genomic processes.

### The sequence of the nucleosomal DNA

The structural, mechanical and functional properties of nucleosomes have also been suggested to depend on the underlying DNA sequence due to the influence of the latter on nucleosome positioning (Widom 2001). This notion has provoked high interest in investigating nucleosomal DNA sequences to identify weak and strong nucleosomes and has led to the development of different artificial sequences which have become widely used in in vitro studies (Trifonov and Nibhani 2015). Recently, the influence of DNA sequence on nucleosome structure was studied using a single-molecule approach combining smFRET and OT (Ngo et al. 2015). This assay allowed the simultaneous manipulation and observation of the nucleosome to probe force-induced local conformational changes. Nucleosomes were found to disassemble by asymmetric and directional unwrapping under force, whereby the relative stiffness of different regions of the nucleosomal DNA dictated the unwrapping direction, with a preference for starting from the stiffer side. When the DNA exhibited similar flexibility on both sides, nucleosomes unwrapped stochastically from either side. Both ends further showed an interplay in which the opening of one end stabilized the other, indicating that even small differences in DNA flexibility on either side could lead to an asymmetric stability of the nucleosome. These findings clearly demonstrate the influence of local DNA flexibility, caused by its sequence composition, on nucleosome stability and DNA accessibility. In a more general context, they suggest a new mechanism contributing to the regulation of DNA accessibility by the nucleosomal DNA sequence and its modifications. However, if and to what extent sequence-dependent effects on nucleosomal DNA dynamics play a role for in vivo processes awaits future investigation, which will likely be challenging, as many different mechanisms cooperate to influence nucleosome (re)organization in the cell.

### Post-translational modification of histones

One well-established mechanism that substantially influences nucleosome structure and dynamics is the post-translational modification (PTM) of the unstructured histone tails protruding from the nucleosome core complex at specific positions (Bowman and Poirier 2015). This form of dynamic chemical alteration of the histones mediated by a great number of dedicated enzymes has been shown to change the structural and dynamic properties of nucleosomes by affecting histone–DNA or histone–histone interactions. The best-studied chemical modifications include histone acetylation and phosphorylation. Their effects on nucleosome structural dynamics were studied recently by complementing different biochemical assays with smFRET (Brehove et al. 2015). Phosphorylation of tyrosine 41 and threonine 45 of histone H3 located in the nucleosomal core near the DNA entry–exit sites was found to enhance DNA accessibility by threefold, as did histone acetylation of lysine 56 in the same region. Remarkably, simultaneous phosphorylation and acetylation were observed to increase DNA accessibility by an order of magnitude. Although DNA accessibility was tested indirectly by a protein-binding assay, which does not allow direct quantification of the intrinsic dynamics of nucleosomes, the study still clearly demonstrates the significant effect of PTMs on DNA unwrapping dynamics. In a broader context, these results suggest that particularly PTMs of the globular domains of the histones have the ability to directly affect nucleosome stability by impacting histone–DNA interactions while modulating the ability of the nucleosome to bind regulatory factors. A large and increasing number of identified PTMs awaits further study at the single-molecule level to advance our understanding of nucleosome structure and dynamics (Arnaudo and Garcia 2013).

### ATP-dependent remodeling

Complementary to the chemical modification of histones, another mechanism that affects the stability and dynamics of nucleosomes is mediated by enzymes that actively reorganize nucleosome structure. These ATP-dependent chromatin remodelers catalyze changes in nucleosome position and composition by inducing nucleosome sliding or (partial) disassembly/assembly of histones upon ATP-hydrolysis (Clapier and Cairns 2009). The nucleosome remodeling process by the ATP-dependent chromatin assembly and remodeling factor (ACF) that contains a catalytic subunit belonging to the imitation switch (ISWI)-family and generates uniformly spaced nucleosomal arrays was recently studied in molecular detail using smFRET (Hwang et al. 2014). This approach allowed for the time-dependent observation of DNA translocation upon remodeling by ACF. Both the linker DNA and the histone H4 tail were found to affect DNA translocation by increasing the pause durations in the remodeling process. The catalytic and accessory subunit of ACF, Snfh2 and Acf1, respectively, were observed to cooperate in detecting the linker DNA with the help of the histone H4 tail. For short linker DNA lengths, Acf1 preferably bound to the N-terminal region of the histone H4 tail, which resulted in autoinhibition of the ATPase activity of Snfh2 while possibly increasing pause durations. With increasing linker DNA lengths, however, Acf1 changed its binding preference towards the linker DNA by releasing the histone H4 tail, which in turn relieved the autorepression mechanism and resulted in activation of the Snf2h ATPase. These results indicate that DNA linker length and the histone H4 tail are important components of nucleosome remodeling by enzymes of the ISWI-family and suggest a potential regulatory mechanism to direct nucleosome spacing. Many chromatin remodelers of different families have been identified, and the details of their remodeling mechanism still need to be studied (Hota and Bartholomew 2011). As demonstrated by the results of this study, single-molecule techniques are highly suitable for this purpose. The bigger challenge seems to be the investigation of all mechanisms affecting nucleosome structure and dynamics in combination.

### Genome processing enzymes

In addition to the mechanisms presented above that specifically target nucleosomes, non-specific external events mediated by other factors influence nucleosome structure and dynamics. Certain enzymes have to exert forces and torques in order to perform their tasks in processing the genome. In a recent study, the effect of force and torque on nucleosome structure was investigated using an angular optical trapping method called the optical torque wrench (OTW) (Sheinin et al. 2013). In addition to allowing the manipulation of biomolecules by means of force, this OT-based technique allows the application and measurement of torque (La Porta and Wang 2004). Qualitatively, torque was found to only have a modest effect on nucleosome disassembly. The unwrapping of nucleosomes always followed a distinct two-step pattern, namely, a sudden release of nucleosomal DNA at lower forces (<6 pN), attributed to the outer turns around the H2A/H2B dimers, and another release at higher forces (≥6 pN), assigned to the inner turn around the (H3–H4)_2_ tetramer. This interpretation and the details of nucleosome unwrapping, however, might need to be reconsidered after the recent observation of asymmetric unwrapping reported above. Quantitatively, however, torque was observed to significantly affect the disruption forces by stabilizing the outer turns and destabilizing the inner turn. Remarkably, the application of positive torque additionally led to a striking loss of H2A/H2B dimers, whereas the (H3–H4)_2_ tetramer remains stably bound to the DNA. These findings suggest a potential role of torque and supercoiling in regulating DNA-templated processes by facilitating the removal of the H2A/H2B dimers. More recently, the effect of supercoiling on nucleosome structure was investigated using AFM and FCS (Elbel and Langowski 2015). The architecture of the nucleosomes was revealed by AFM imaging, while their stability was studied by measuring the diffusion constants upon salt-induced destabilization using FCS. Nucleosome structure was found to be dependent on the sign and density of the superhelical turns. Negative supercoiling resulted in more compact and stable nucleosomes that were resistant to changes in salt concentration. In contrast, nucleosomes reconstituted on either relaxed or positively supercoiled DNA were observed to be more open and prone to salt-induced disassembly. Destabilization of these nucleosomes, leading to the enhanced eviction of the H2A/H2B dimers, was observed to start at ∼600–800 mM monovalent salt concentration. The (H3–H4)_2_ tetramer, however, was found to dissociate later at salt concentrations of >1000 mM. These results from the combined approach of imaging and fluorescence spectroscopy clearly demonstrate the significant impact of DNA topology on nucleosome structure and stability and further support the notion of DNA supercoiling as a potential mechanism for regulating the genome by facilitating histone eviction. Investigating structural transitions in the histone octamer itself under torque appears to be a logical next step to reveal more details of the force- and torque-induced disassembly of nucleosomes. The single-molecule approaches, however, need to take into account the biological relevance of all components, such as the range of the applied forces and torques, as well as the salt concentrations used. The regular observation of distinct behavior for the H2A/H2B dimers and the (H3–H4)_2_ tetramer raises more questions about the architecture and dynamics of subnucleosomal structures which will be discussed in the next section.

## Subnucleosomal structures and dynamics

As mentioned above, nucleosomes have been observed to lose their outer H2A/H2B dimers under force, torque or changes in salt concentration, while (H3–H4)_2_ tetramers remain bound to the DNA. These findings indicate the existence of intermediate nucleosome states, several of which have in fact been reported in different studies (Andrews and Luger 2011; Lavelle and Prunell 2007; Luger et al. 2012; Zlatanova et al. 2009). The assembly of nucleosomes happens in a stepwise manner through the initial binding of the (H3–H4)_2_ tetramer to the DNA, followed by the incorporation of the two H2A/H2B dimers (Polo and Almouzni 2006). In the absence of DNA but under otherwise physiological conditions, the histone octamer itself dissociates into the (H3–H4)_2_ tetramer and H2A/H2B dimers (Luger 2001). Specific proteins called histone chaperones exist to bind and stabilize histones and to control their interactions for the assembly or disassembly of nucleosomes (Gurard-Levin et al. 2014). These proteins can also alter the composition of nucleosomes by being involved in the replacement of core histones with histone variants which differ in the protein sequence and can affect both histone–DNA and histone–histone interactions to specifically change nucleosome structure and dynamics (Talbert and Henikoff 2010). In addition, some histone chaperones contribute to the removal of H2A/H2B dimers during transcription, such as the facilitates chromatin transcription (FACT) complex (Reinberg and Sims 2006). Therefore, a reorganization of nucleosomes into substructures seems plausible, even crucial, in the context of chromatin dynamics for regulating DNA-templated processes. Thus, investigating subnucleosomal particles can provide further insight into the structure and dynamics of full nucleosomes.

As a whole, nucleosomes have been found to undergo a conformational transition upon positive torsional stress by changing their ‘chirality’ from a left-handed to a right-handed DNA-wrapping into a reversed nucleosomal structure called reversome (Bancaud et al. 2007). Another nucleosome conformation, termed the split nucleosome, was observed in the form of partial splitting of the H2A/H2B dimers from the (H3–H4)_2_ tetramer while remaining bound to the DNA during salt-induced disassembly, with an eventual stepwise release of the H2A/H2B dimers and the (H3–H4)_2_ tetramer (Bohm et al. 2011). Also, passage of the transcription enzyme RNAP II through the nucleosome was found to produce a hexameric subcomplex missing one H2A/H2B dimer, termed a hexasome (Bintu et al. 2011; Kireeva et al. 2002). The subnucleosomal structure that only contains the (H3–H4)_2_ tetramer is called a tetrasome (Alilat et al. 1999). Tetrasomes were then also found to have the additional, remarkable feature of intrinsically switching between a left-handed and a right-handed ‘chirality’. Thus, tetrasomes constitute an important subnucleosomal structure to study.

**Fig. 6:**
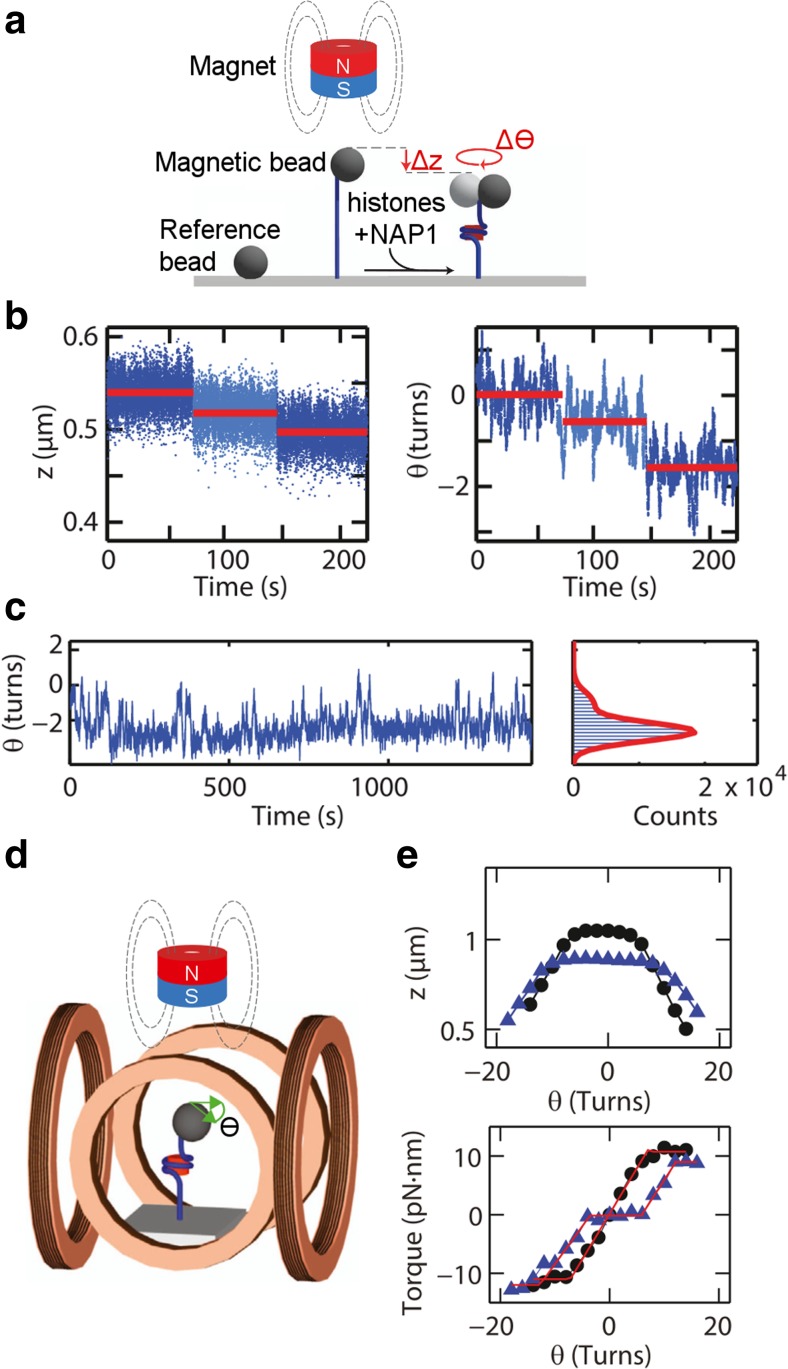
Real-time assembly and structural dynamics of tetrasomes. **a** A DNA molecule is tethered between a glass coverslip and a magnetic bead trapped by a cylindrical magnet, thereby allowing its free rotation. The injection of histones together with nucleosome assembly protein 1 (*NAP1*) yields histone assembly reflected by a decrease in the tether’s extension (*z*, in μm) and the rotation angle (*θ*, in turns), which is related to the tether’s twist and writhe (linking number). **b** The extension and angle time traces show instantaneous changes in a stepwise manner upon tetrasome assembly. **c** The angle time traces of assembled tetrasomes reveal frequent transitions between two distinct linking numbers corresponding to a structural change in their handedness. **d** Two pairs of Helmholtz-coils are used to generate a horizontal magnetic field that is rotated by alternating the applied current to generate precisely controlled torques. **e** The rotation–extension traces of DNA molecules containing tetrasomes (*blue triangles*) show smaller extensions and broadening compared to the traces obtained with bare DNA (*black circles*), indicating assembled tethers and torque absorption, respectively. Torque absorption is verified by the rotation–torque curves revealing an additional plateau for small torques (in pN∙nm) applied to tetrasomes (*blue triangles*) that is absent with bare DNA (*black circles*). All panels (**a**–**e**) are figures reprinted or adapted from Vlijm et al. (2015a), Copyright (2015), with permission from Elsevier. This figure is not included in the present article’s Creative Commons license

The nucleosome assembly protein 1 (NAP1)-mediated assembly of full nucleosomes or tetrasomes was recently investigated in real-time using freely-orbiting magnetic tweezers (FOMT) and electro-magnetic torque tweezers (eMTT) (Vlijm et al. 2015a). These novel MT techniques enable the study of the dynamics and the impact of small, and well-controlled torques on biomolecules (Janssen et al. 2012; Lipfert et al. 2011). In this study, a bare double-stranded DNA (dsDNA) molecule was tethered between the glass coverslip and a magnetic bead that was trapped by a permanent magnet of cylindrical form, with the bead allowed to freely rotate on a circular trajectory (Fig. 6a). Upon injection of the histones with NAP1, nucleosome assembly occurred instantaneously in steps that were reflected both in the tether’s extension as well as the rotation angle, corresponding to the tether’s twist and writhe (linking number). Nucleosome formation was also achieved by first assembling tetrasomes followed by the incorporation of the H2A/H2B dimers. Interaction with the DNA was not observed for H2A/H2B dimers, whereas they readily bound to previously formed tetrasomes. This observation again confirms the necessity of tetramer binding before the additional incorporation of H2A/H2B dimers. (H3–H4)_2_ tetramers assembled instantaneously onto the DNA and remained stably bound for long times, indicating that tetrasomes are viable nucleosomal substructures (Fig. 6b). Remarkably, tetrasomes were further found to spontaneously switch their ‘chirality’ between a preferred left-handed and a less frequently occurring right-handed DNA wrapping, which is referred to as ‘handedness flipping’ (Fig. 6c). These structural dynamics may explain the significantly delayed accumulation of torque in the DNA tether containing tetrasomes in torque measurements (Fig. 6d, e). The conversion of tetrasomes from one chirality state to the other by applying weak torques was suggested as the underlying mechanism for this phenomenon. In contrast, this sort of structural dynamics and behavior was not observed for nucleosomes. On the whole, this study provides new insights into the structural dynamics of nucleosomes in the context of substructures, suggesting a potential mechanism to regulate supercoiling during DNA-templated processes by absorbing the generated torque. Very similar results were obtained in a more recent follow-up study with the histone variant H3.3 (Vlijm et al. 2015b). Comparable details of the nucleosome assembly and the structural dynamics of tetrasomes containing the histone variant H3.3 indicate that the incorporation of this variant histone, which in the cell occurs upon histone loss in processes such as transcription, does not give rise to changes in nucleosomal structure and dynamics, but rather may affect other processes such as the recruitment of specific histone chaperones or remodelers.

The dynamics of (sub)nucleosomal structures were also investigated using high-speed-AFM (HS-AFM) (Katan et al. 2015). This novel technique enables the visualization of the structure and dynamics of biomolecules at acquisition rates of up to 10 Hz or higher (Ando et al. 2013). The histone–DNA complexes were either reconstituted by salt dialysis or assembled using NAP1 and deposited onto a mica substrate for incubation prior to imaging in liquid. Nucleosomes were found to spontaneously disassemble in a fast process on a timescale of 1 s, while tetrasomes underwent several different dynamic changes, such as sliding, hopping between two stable positions involving a change in the ‘handedness’ of the DNA-wrapping and disassembly with the concomitant formation of a DNA-loop that remains stable for minutes. In addition to illustrating the suitability of HS-AFM for probing DNA–protein interactions, this study reveals the highly dynamic nature of (sub)nucleosomal structures which may add an additional layer of flexibility in the accommodation and control of processes such as transcription, replication and repair.

Overall, all nucleosomal (sub)structures and their properties carry significant biological potential in the context of gene regulation during essential cellular processes. There are still many questions left to be answered on their function which could also be explained in the context of other nucleosome-related mechanisms. Single-molecule techniques are a promising tool to advance the research on this topic by the development and application of more complex assays, as described in the next section.

## The nucleosome as a barrier

As mentioned above, nucleosomes can act as dynamic mechanical barriers to related DNA-binding proteins and DNA-processing enzymes. Many different processes that influence nucleosome structure, such as intrinsic dynamics and remodeling involving histone chaperones, chromatin remodeling enzymes as well as post-translational modifications, possibly facilitate the overcoming of the barrier. However, the exact mechanisms underlying genome processing through nucleosomes that reveal the fate of colliding enzymes and histones still remain unclear. As the first step of gene expression and one of the crucial processes to maintain cell viability and function, transcription has become a topic of great interest in the context of nucleosome research at the single-molecule level as well (Dangkulwanich et al. 2014; Teves et al. 2014).

Mimicking genome processing by unzipping dsDNA molecules containing single nucleosomes using OT revealed the locations and features of histone–DNA interactions at ∼1-bp resolution (Hall et al. 2009). The 5-bp periodicity of these strong interactions within three broad regions indicates that nucleosomes actually represent a considerable energy barrier to DNA-processing enzymes. This conclusion was further confirmed by several direct studies of transcription through nucleosomes using purified RNAP II in different assays based on the common single-molecule techniques (Bintu et al. 2011, 2012; Hodges et al. 2009; Jin et al. 2010). Nucleosomes were found to have a significant effect on the dynamics of RNAP II by locally increasing the density and duration of its pausing, as well as by decreasing their actual (pause-free) velocity (Hodges et al. 2009). The authors concluded that the changes in polymerase dynamics are governed by fluctuations in nucleosome unwrapping, which would either deny or give the polymerase access to nucleosomal DNA in the closed or open nucleosome conformation, respectively. In addition to increasing the pause density and duration, nucleosomes were also observed to induce backtracking of polymerases. A successive RNAP was, however, found to release the preceding polymerase from backtracking to restart and even continue with elongation at a higher rate (Jin et al. 2010). This finding suggests that multiple RNAP II enzymes could cooperatively increase transcription efficiency. The nucleosomal barrier to transcription was further shown to be highly controlled by specific structural elements of the nucleosome (Bintu et al. 2012). Elimination of the histone tails and destabilization of specific histone–DNA interactions enabled transcription to overcome the nucleosomal barrier more easily. The greater efficiency of transcription observed for weakened histone–DNA interactions shows their essential role in nucleosome stability, while, alternatively, the histone tails could have a significant function in the recruitment and mode of action of specific remodelers. Some details of nucleosomal fate during transcription were revealed by simultaneous imaging of RNAP and nucleosomes at different stages of transcription using AFM (Bintu et al. 2011). While some nucleosomes did not change their position upon transcription, others were found upstream of their initial location, which was explained by a DNA-looping mechanism for histone transfer. In addition, some of the transcribed nucleosomes showed a smaller size depending on the elongation rate, which was ascribed to the loss of one H2A/H2B dimer during transcription resulting in the formation of a hexamer. These studies have convincingly illustrated how transcription through nucleosomes both requires and causes structural changes that may occur by RNAP-mediated changes in supercoiling and/or the action of the accessory factors, such as histone chaperones, chromatin remodelers and other transcription factors.

Single-molecule research on the transcription of nucleosome substrates is now moving towards more complex systems involving additional factors. The effect of nucleosomes on the binding and dissociation of transcription factors (TF) was recently studied using fluorescence microscopy, including smFRET (Luo et al. 2014). In this study, dsDNA molecules containing a binding site for either the TF LexA or the TF Gal4 were used to reconstitute nucleosomes which remained intact or were trapped in an open conformation upon TF binding. Monitoring the FRET efficiency allowed the TF binding and dissociation rates to be determined. Nucleosomes were not only found to decrease the binding rate of the TFs by ∼500-fold, but also to significantly increase their dissociation rate by ∼1000-fold compared to bare dsDNA molecules. These results show that nucleosomes regulate TF access to DNA and propose a possible mechanism for facilitating TF exchange. This regulatory function of the nucleosome may also apply to other DNA-binding proteins.

**Fig. 7:**
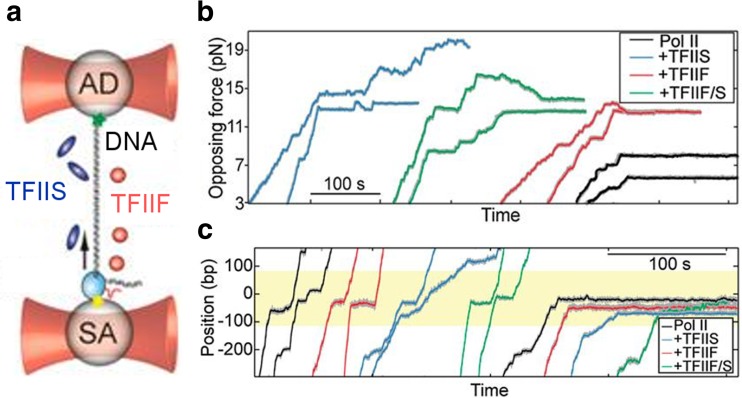
Real-time dynamics of transcription enzyme RNA polymerase II (RNAP II) elongation activity in the presence of transcription elongation factors (TFs). **a** A DNA molecule is attached to an anti-digoxigenin (AD)-coated bead at its downstream end and tethered to another streptavidin (SA)-coated bead through an RNAP II complex with two optical traps. Two different TFs (*TFIIS*, *blue*; *TFIIF*, *red*) are injected to investigate their effect on transcription dynamics. **b** Time traces of the opposing force (in pN) to the transcribing RNAP II complex reveal facilitated elongation by the TFs individually (*TFIIS*, *blue*; *TFIIF*, *red*) or together (*TFIIF/TFIIS*, *green*) compared to RNAP II alone (*black*). **c** Time traces of the RNAP II position (in bp) along a DNA molecule containing an initially well-positioned single nucleosome (*yellow-shaded band*) show stimulation of RNAP II processivity (*black*) through nucleosomes by the TFs individually (*TFIIS*, *blue*; *TFIIF*, *red*) or together (*TFIIF/S*, *green*). All panels (**a**–**c**) are figures reprinted with minor changes from Ishibashi et al. (2014), with permission from PNAS. This figure is not included in the present article's Creative Commons license

In another study, the effect of two general transcription elongation factors, TFIIS and TFIIF, on the transcription process of both bare and nucleosomal DNA was investigated using OT (Ishibashi et al. 2014). The specific assay comprised a dsDNA molecule directly attached to one bead at its downstream end and tethered to another bead through an RNAP II complex (Fig. 7a). Depending on the orientation of the DNA molecule, either an opposing or an assisting force could be applied to the RNAP, allowing for complementary insights into the transcription dynamics. Both TFs were added independently or together and were not found to significantly influence the actual (pause-free) elongation rate. However, the TFs did affect the pausing dynamics of the RNAP in a distinct manner (Fig. 7b). TFIIS reduced the pause duration, while TFIIF decreased both the pause density and duration depending on the force. The same effects also enhanced the progression of RNAP through the nucleosomal barrier (Fig. 7c). These single-molecule experiments thus helped to pin down the details on the dynamics and the biophysical mechanisms by which TFIIS and TFIIF enact their known positive effects on RNAP II elongation efficiency.

In a very recent study, the effects of nucleosome remodelling by either yeast SWI/SNF or ISW1a on the bound TF Gal4DBD and vice versa were investigated using the above-mentioned OT-based unzipping technique (Li et al. 2015). In this assay, the single-stranded DNA (ssDNA) segments of an already partially unzipped dsDNA molecule containing a single nucleosome and a bound TF were tethered between the glass coverslip and a microsphere. The molecules were further unzipped by moving the glass coverslip away from the bead, and the strengths and locations of the nucleosome and the bound TF were simultaneously reflected as peaks in the force-extension traces at ∼1 bp resolution. Unzipping molecules after finalized remodeling reactions with either remodeler revealed that a TF represents a considerable barrier to remodeling by ISW1a for nucleosome repositioning. However, the SWI/SNF remodeler could generate nucleosome sliding accompanied by removal of the TF without a significant effect on nucleosome positioning. Interestingly, the nucleosomes were located in opposite directions after remodeling, indicating that both remodelers have distinct roles in nucleosome positioning in the presence of TFs. This study demonstrates a potential mechanism for the regulation of nucleosome remodeling by TFs and vice versa, which could further have a major role in the regulation of transcription in general.

In summary, the latest advances of in vitro single-molecule research on transcription involving nucleosomes represent a successful next step towards understanding more details of this complex process. The combination of multiple proteins and mechanisms in a crowded environment seems a promising approach to elucidate additional details of this complex fundamental process. The further development of this kind of assays is expected to also finally explain the still striking feature of transcription through chromatin in vivo occurring at similar rates to in vitro transcription of bare DNA (Izban and Luse 1992).

## Conclusions and future perspectives

As the basic packaging unit of chromatin, the nucleosome represents a fundamental DNA–protein complex whose study is required for understanding the organization and regulation of the genome during essential cellular processes. In this context, nucleosomes are not static, but highly ordered and dynamic entities. Their structure and dynamics are continuously altered by different mechanisms involving spontaneous conformational changes, the properties of the underlying DNA, post-translational modifications of the histones, ATP-dependent remodelers, external forces and torques, the incorporation of histone variants, and interactions with histone chaperones and other related proteins.

In this review, we have introduced the most commonly used single-molecule techniques in nucleosome research and presented recent insights they have provided into nucleosome structure, function and dynamics. Studies of individual nucleosomes in a time-resolved manner have revealed transient conformational states on fast timescales, such as breathing and possibly gaping. The structural and chemical properties of the nucleosomal DNA are suggested to influence nucleosome structure and dynamics. Post-translational modifications of histones have been found to significantly affect the intrinsic dynamics and the stability of nucleosomes by altering DNA–histone or histone–histone interactions. More assays have been developed and extended by using other nucleosome-related components, such as histone chaperones and remodeling enzymes, to study their underlying molecular mechanisms and impact on nucleosome (dis)assembly, architecture and dynamics. Various subnucleosomal structures have been identified both in vivo and in vitro and are believed to play an important role in the regulation of the genome during nuclear processes. Some great insights into the function and fate of the nucleosome as a barrier during DNA-templated processes have been provided by the incorporation of genome processing machineries. Single-molecule studies in the context of histone variants and replication could provide more insights into (sub)nucleosomal structure and dynamics and possibly reveal new mechanisms to complement current knowledge.

**Fig. 8:**
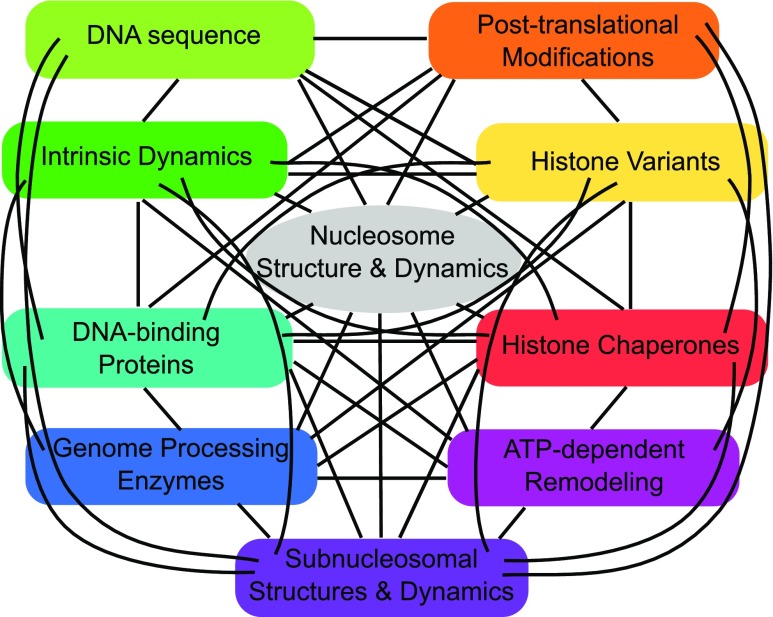
Overview of mechanisms influencing nucleosome structure and dynamics. Nucleosomes are intrinsically dynamic (*dark green*) entities, and their structure and dynamics are additionally affected by many different mechanisms, such as sequence and chemical properties of the underlying DNA (*light green*), post-translational modifications of histones (*orange*), the incorporation of histone variants (*yellow*), the interactions with histone chaperones (*red*), ATP-dependent remodelling (*magenta*), the reorganization in subnucleosomal substructures (*violet*) and non-specifc external factors like genome processing machineries (*dark blue*) and DNA-binding proteins (*blue/green*). All these mechanisms are coherent and act in a concerted manner (*black lines*)

However, as several different mechanisms involving various proteins simultaneously act on nucleosomes in a concerted manner, many questions remain to be answered regarding the details of their interplay (Fig. 8). A powerful approach seems to be the combination of fluorescence and force spectroscopy techniques, which has already been used to probe various DNA–protein interactions (Cordova et al. 2014). Further advances in microscopy and sample preparation techniques at the single-molecule level will enable measurements in crowded conditions involving several different components and mechanisms. This will allow the recapitulation of actual biological processes in an environment that approaches in vivo conditions. In the vast majority of single-molecule methods for studying nucleosomes, specifically designed and immobilized DNA constructs containing strong nucleosome-positioning sequences, purified, engineered or labeled proteins and non-physiological buffers are used. This allows for a better control of the biological system, but in the end does not reflect the native situation. On the other hand, in vivo single-molecule studies that are mainly based on fluorescence microscopy have been very challenging due to specific treatments of live cells and in particular the low spatial resolution. However, recent developments in live cell microscopy towards super-resolution microscopy with highly improved spatial resolution hold great promise for studies of chromatin structure and dynamics in vivo (Lakadamyali and Cosma 2015). Collectively, the complementary insights from different techniques and assays will allow researchers to put individual parts of the huge puzzle on chromatin structure and dynamics together to advance our understanding of one of the fundamental aspects of life.
